# Common pathways and functional profiles reveal underlying patterns in Breast, Kidney and Lung cancers

**DOI:** 10.1186/s13062-021-00293-8

**Published:** 2021-05-26

**Authors:** Sergio Romera-Giner, Zoraida Andreu Martínez, Francisco García-García, Marta R. Hidalgo

**Affiliations:** 1 Bioinformatics & Biostatistics Unit, Principe Felipe Research Center, 46012 Valencia, Spain; 2 ATOS Research & Innovation (ARI), 28037 Madrid, Spain; 3 Foundation Valencian Institute of Oncology (FIVO), 46009 Valencia, Spain; 4 Spanish National Bioinformatics Institute, ELIXIR-Spain (INB, ELIXIR-ES), 46012 Valencia, Spain

**Keywords:** Cancer, Signaling, Pathways, Functional analysis, Survival, miRNAs, Artificial intelligence

## Abstract

**Background:**

Cancer is a major health problem which presents a high heterogeneity. In this work we explore omics data from Breast, Kidney and Lung cancers at different levels as signalling pathways, functions and miRNAs, as part of the CAMDA 2019 Hi-Res Cancer Data Integration Challenge. Our goal is to find common functional patterns which give rise to the generic microenvironment in these cancers and contribute to a better understanding of cancer pathogenesis and a possible clinical translation down further studies.

**Results:**

After a tumor versus normal tissue comparison of the signaling pathways and cell functions, we found 828 subpathways, 912 Gene Ontology terms and 91 Uniprot keywords commonly significant to the three studied tumors. Such features interestingly show the power to classify tumor samples into subgroups with different survival times, and predict tumor state and tissue of origin through machine learning techniques. We also found cancer-specific alternative activation subpathways, such as the ones activating STAT5A in ErbB signaling pathway. miRNAs evaluation show the role of miRNAs, such as mir-184 and mir-206, as regulators of many cancer pathways and their value in prognoses.

**Conclusions:**

The study of the common functional and pathway activities of different cancers is an interesting approach to understand molecular mechanisms of the tumoral process regardless of their tissue of origin. The existence of platforms as the CAMDA challenges provide the opportunity to share knowledge and improve future scientific research and clinical practice.

**Supplementary Information:**

The online version contains supplementary material available at 10.1186/s13062-021-00293-8.

## Background

Cancer is a major health problem that represents the second cause of death due to disease worldwide after cardiovascular diseases. Aging, population growth, cancer heterogeneity, as well as the bad prognosis of some cancers when detected late or the lack of effective treatments, among other factors, contribute to these numbers [1]. Therefore, research to improve diagnostic and predictive tools for early detection and new treatment strategies is crucial to reduce cancer mortality rates.

Until recently, cancer diagnosis and treatment were mostly faced through an histologic point of view: scientists inferred different aspects of cancer, such as tumor grading and malignancy, by the comparison of cells found in tumoral and healthy tissue through microscopy techniques [2]. This trend continues today, using the vast array of histologic images available as training input for artificial intelligence models for accurate cancer diagnosis and early tumor detection [3]. Still, this approach is limited due to the complexity and invasiveness of tissue extraction by biopsies from patients and the difficulties of certain tissues to undergo medical imaging analysis.

In the last decades, biotechnological advances have allowed the scientific community to study cells from another perspective: that of their genes, proteins and metabolites. This new approach, designed as *omics* sciences, allows us to delve deeper into cells inner mechanisms, their regulation and the alterations that differentiate a tumoral cell from a healthy one [4]. As a result, the omics data complement histological findings, helping to further characterize tumors and improving diagnosis and treatment.

The further advances in high-throughput, next generation sequencing technologies, Big Data and bioinformatic tools even allow the combination of different aspects of these omics (such as genomics, proteomics or metabolomics). From this combination, we are able to model the gene and protein interaction networks which allow the cell to perform its multiple functions: the signaling pathways [5, 6]. These pathways and their alterations are closely related to cancer, for what they are growing in importance as an asset for better understanding of cancer mechanisms, causes and survival [7, 8].

Another important finding regarding cancer mechanisms comes from the microRNAs and their role in gene expression regulation [9]. MiRNAs work in a post-transductional level: in animals, they bind into a target gene’s mRNA, inhibiting the translation to protein. The action of these miRNAs over critical genes, such as oncogenes or tumor suppressor genes, is heavily related with cancer cells conversion and development. As a result, miRNA study and its specific relation with different types of tumors is becoming a pivotal field of research.

Nonetheless, despite what has been described in previous paragraphs, a great amount of omic information doesn’t translate directly to improvements to the patient [4]. It constitutes a challenge to process and research on these data to fully understand cancer complexity. Regarding this, the initiatives to put this kind of biological data into the hands of the scientific community in order to provide new medical and biological insights are a key part for future advances. One of these initiatives are the challenges posed by the Conference on Critical Assessment of Massive Data Analysis (CAMDA).

The goal of the CAMDA open challenges is to reach novel solutions or methodologies to better understand omics complex data. In particular, the CAMDA 2019 Hi-Res Cancer Data Integration Challenge goal was to gain new biological insights based on cancer data provided by Genomic Data Commons (GDC) [10]. This data was comprised of human genomic samples of two well-documented cancer types, Breast Cancer (BRCA) and Lung Adenocarcinoma (LUAD), and another which is less well studied, Kidney Renal Clear Cell Carcinoma (KIRC), with their corresponding levels of miRNAs.

In this work we present our approach to the CAMDA 2019 Hi-Res Cancer Data Integration Challenge, consisting of a characterization of the common deregulated pathways and functions across breast, lung and kidney cancers. The objective of the work is to assess the common functional patterns in the three cancers to allow a better comprehension of the cancer mechanisms, and help in the development of future common treatments. We also assess whether the inherent cancer differences found at the common pathways and functions are solid enough to classify these three types of cancer correctly and look for underlying mechanisms that could be used to target diagnosis or treatment based on cancer.

For this task, we use the pathway analysis tool Hipathia [11], which allows the interpretation of transcriptomics data at a pathway and functional level, transforming the transcriptional values of genes into pathway and molecular functions activation, using Uniprot protein functions [12] and Gene Ontology molecular terms [13, 14]. We also relate miRNA activity with the common pathways found, showing interesting activity correlations. Finally, we check out the quality of the resulting pathway activity dataset as an input for simple machine learning model classificators.

Our results point to a group of common pathways and functional terms suitable to define and cluster BRCA, KIRC and LUAD cancers into groups with different survival times. The miRNA analysis revealed their regulation role on the pathways studied and their relevant potential as prognostic factors. We also found specific pathways and functional terms that may be key in underlying molecular mechanisms for each cancer type. Finally, our trial in machine learning model classification is promising for the development of artificial intelligence models, further in depth studies, that could guide clinical practice.

## Results

### Tumor vs. normal tissue comparisons

The datasets from BRCA, KIRC and LUAD were downloaded and processed as described in methods, and the comparison of tumor vs. normal samples for the levels of activity of the pathways and functions analyzed were applied accordingly. In Hipathia, each signaling pathway is subdivided in a series of subpathways. These subpathways represent a specific path which links an input node (such as cells receptors) to a final effector protein, and from now on will be referred to as *paths* for the sake of brevity.

**Fig. 1:**
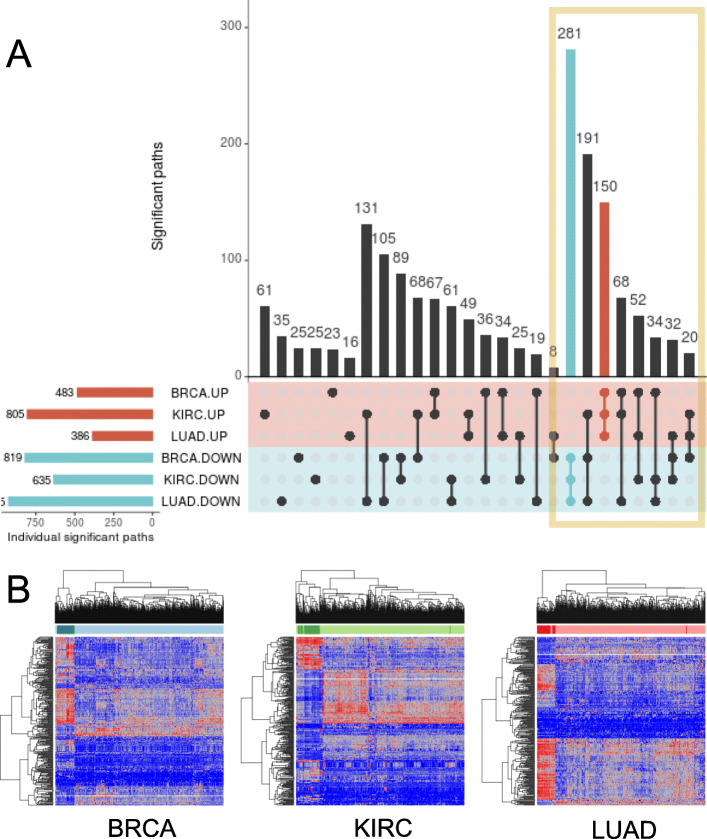
Graphical analysis of the common path values across the three cancers. **a**: Upset plot representing the number of coincident significant paths between cancers. For each cancer type, two groups have been created: the group of the up-activated paths (denoted by UP), and the group of the down-activated paths (denoted by DOWN). Therefore, the same path can not be at the same time in the same cancer’s UP and DOWN groups. Red and blue horizontal bars represent the number of significant paths in each group. Each vertical bar in the plot represents the intersection of the groups in the inferior rows with a solid point, and the exclusion of the groups in the inferior rows with a shaded point. An orange box surrounds the part of the UpSet plot representing the paths which are significant in all three cancers. The blue and red vertical bars represent the paths which are simultaneously down- and up-regulated in the three cancers, respectively. **b**: Heatmaps of the significantly common path values, represented inside the orange box of the UpSet plot above. Samples and paths were ordered following the results of a hierarchical clusterization. Tumor samples are colored in blue while normal tissue samples are colored in light blue. In the heatmap, higher activation path values are colored in red and lower activation path values in blue. Left: BRCA cancer data. Center: KIRC cancer data. Right: LUAD cancer data

The comparison of the different activities, at the path and functional level, between tumor and healthy tissue samples returned the number of up- and down-activated significant features in each cancer, shown in Table 1. The number of coincident significant up- or down-activated paths among the 3 cancers is shown in Fig. 1a. Supplementary Figures SF1 and SF2 reproduce the scheme in Fig. 1 for Uniprot and GO terms values.

**Table 1:** Number of significant results per cancer and feature

	Paths			Gene Ontology			Uniprot		
	UP	DOWN	TOTAL	UP	DOWN	TOTAL	UP	DOWN	TOTAL
**BRCA**	483	819	1302	388	848	1236	32	93	125
**KIRC**	805	635	1440	804	541	1345	51	65	116
**LUAD**	386	925	1311	242	1165	1407	27	96	123

### Common features in BRCA, KIRC and LUAD cancers

After the tumor versus normal tissue analysis, we focus on the commonly significant features across the three cancers, from now on *common* features. Features presenting also a common sign of the logFC in all three cancers were categorized as *unidirectional* (changes are always in the same direction: up or down activated), and those with a different sign of the logFC as *bidirectional*.

There exist two types of unidirectional features (all UP or all DOWN changes), and 6 types of bidirectional features, depending on the UP and DOWN pattern across the three cancers. The number of common, unidirectional and bidirectional features is summarized in Table 2, and the number of specific unidirectional and bidirectional subtypes is shown in the orange box of Fig. 1a.

**Table 2:** Total number of features, summarized by their directionality

Feature	Common			Not common	Total analyzed
	Unidirectional	Bidirectional	Total		
Paths	431	397	828	1040	1868
GO Terms	400	512	912	742	1654
Uniprot Keywords	52	39	91	51	142

For further reference, Supplementary Tables S1, S2 and S3 in supplementary material show the list of pathways, GO terms and Uniprot keywords, respectively, their common, unidirectional or bidirectional status and the *p-values* of the comparisons between tumor and normal samples in each of the cancer types, ordered by the sum of the negative logarithm of the three *p-values*.

### Common significant paths in BRCA, KIRC and LUAD cancers

We have found 828 statistically significant paths common to BRCA, LUAD and KIRC cancers. The values of the common paths are depicted as heatmaps in Fig. 1b. Samples and features were grouped by hierarchical clustering, and clear patterns emerged from the grouping in the three cancer studies, allowing for an easy separation between tumor and normal samples.

As expected, pathways related to tumor growth, metastatic potential, immune evasion and treatment resistance, among others, appear as dysregulated in all three cancer types.

Table 3 shows the top ten most significant paths in all three cancers, after ordering them by the sum of the negative logarithm of the three *p-values*. Paths are represented by the pathway to which they belong and their effector protein. They are displayed with their associated logFC sign in each cancer (UP or DOWN) and the cancer prognosis factor related to their effector protein based on previous literature.

**Table 3:** Top ten most significant paths, their cancer-type sign and prognosis factor according to the literature

Path	BRCA	KIRC	LUAD	Prognosis Factor
PPAR:CD36	DOWN	UP	DOWN	Metastatic potential and immunotherapy resistance [15–17], UP: poor prognosis
Axon Guidance: CFL1	DOWN	DOWN	DOWN	Invasion, metastasis progression, therapy resistance, DOWN: better prognosis
Melanogenesis: TYRP1	UP	DOWN	DOWN	Metastatic potential, DOWN: poor prognosis
Thyroid hormone: RCAN1	DOWN	DOWN	DOWN	Metastatic potential, treatment resistance (sunitinib) [18, 19], DOWN: poor prognosis
Aldosterone synthesis and secretion: PDE2A	DOWN	DOWN	DOWN	Invasive and metastatic potential [20], DOWN: poor prognosis
Aldosterone-regulated sodium reabsorption: FXYD4	UP	DOWN	DOWN	Recurrence-free survival following surgery [21], DOWN: poor prognosis
Aldosterone-regulated sodium reabsorption: SCNN1A	DOWN	DOWN	DOWN	Proliferation, migration, poor prognosis [22, 23], DOWN: better prognosis
Aldosterone-regulated sodium reabsorption: KCNJ1	DOWN	DOWN	DOWN	Prognostic factor of patient’s survival, Metastatic potential [24], DOWN: worse prognosis
Salivary secretion: RYR3	DOWN	DOWN	DOWN	Unfavorable prognosis and upcoming malignant conversion [25], DOWN: poor prognosis
Proteoglycans in cancer: CTNNB1	DOWN	DOWN	DOWN	Unfavorable outcomes, metastasis potential, immunotherapies resistance [26, 27], DOWN: better prognosis

Many of the rest of the common paths are related to the following pathways: Cell cycle (up-activated paths ending in RB1 and protein complexes including MCM and ORC families), Toll-like receptor signaling pathway (up-activated path ending in proteins CXCL9, CXCL10, CXCL11, IFNB1, related to immune response), Hippo signaling pathway (down-activated paths ending in ID1, NKD1 or CTGF related to clinicopathological malignance), MAPK signaling pathway (down-activation of paths ending in NR4A1 and MAP 3 K4, which are reportedly tumor suppressors, and up-activation of pathsending in ELK1, TP53 and CDC25B, with oncogenic properties), PPAR signaling pathway (down-activation of paths ending in proteins AQP7, GK, PCK1, ACAA1, CPT1C, ACSL1, LPL, SLC27A4, strongly related to lipid and fatty acid metabolisms), ERBB signaling pathway (paths ending in proteins CDKN1A, CDKN1B, BAD, GSK3B and EIF4EBP1, involved in checkpoint cellular cycle and oncogenes, are up, and those ending in RPS6KB1, STAT5A and PRKCA involved in proliferation, drug resistance and survival are down) and AMPK signaling pathway.

Interestingly, when exploring differential expression of the genes involved in those paths, cancer-specific patterns arise. As an example, Supplementary Figure SF3A shows the boxplots representing the distribution of the path *AMPK signaling pathway: CCNA2* (the path from the KEGG *AMPK signaling pathway* with effector protein CCNA2) in tumor and normal samples for each of the cancers. A clear common up-activation pattern is observed in tumor samples of three cancer types, but only LEPR and CCNA2 nodes are differentially expressed in the same direction in all three cancers. Yet, the joint path activity presents the same behaviour in all of them. Supplementary Figure SF3B shows the Hipathia visualization for the same path for the tumor vs. normal comparisons in BRCA (top), LUAD (center) and KIRC (bottom), including gene differential expression.

**Fig. 2:**
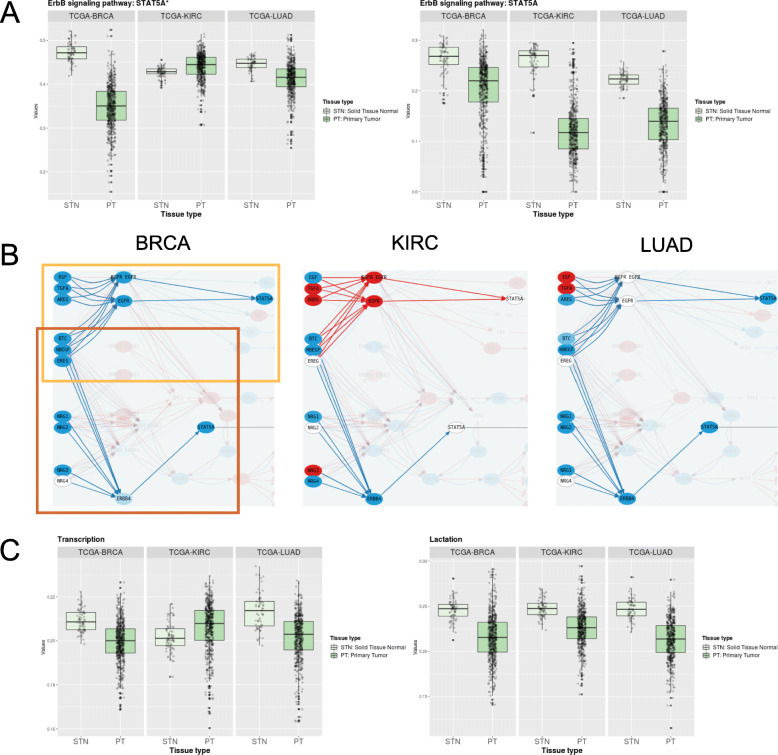
Alternative path activation related to *ErbB signaling pathway:* ***STAT5A****.* **a**: Boxplots of the path values of paths *ErbB signaling pathway: STAT5A* and ErbB signaling pathway: STAT5A* for the tumor and normal tissue samples across the three cancers. **b**: Graphical representation of paths *ErbB signaling pathway: STAT5A* (in the dark orange box) and ErbB signaling pathway: STAT5A* (in the light orange box) including gene differential expression across the three cancers. Red, blue and white nodes correspond to significant up- or down-regulated genes, or non-significant nodes, respectively. Red and blue arrows represent paths significantly up- or down-activated. **c**: Boxplots of the activity values of functions *Lactation* and *Transcription*, which are promoted by paths *ErbB signaling pathway: STAT5A* and ErbB signaling pathway: STAT5A*, for the tumor and normal tissue samples across the three cancers

The case of the STAT5A effector gene in the ErbB Signaling pathway is also paradigmatic. This gene appears in the ErbB signaling pathway as the effector of two different paths, both selected as commonly significant, although with different directional behaviour: while *ErbB signaling pathway: STAT5A* is unidirectional, being down-activated in all three cancers, ErbB signaling pathway: STAT5A* is bidirectional, being up-activated in KIRC and down-activated in BRCA and LUAD, see Fig. 2a. Notice that the effector STAT5A is being activated in each path by different predecessor proteins, see Fig. 2b, orange boxes. Also, different patterns of gene dysregulation are visible depending on the cancer type, see Fig. 2b. STAT5A itself appears as down-regulated in BRCA and LUAD, but is not dysregulated in KIRC. From the cancer mechanisms point of view, on the one hand, the fact that STAT5A is not down-regulated in KIRC may allow this tumor to regulate its behaviour not through up- or down-regulation of the gene itself, but through the dysregulation of the specific genes in each of the paths, tuning gene activity through the path and not the gene.

On the other hand, this mechanism may allow the cancer to select specific functions of a gene to be up-activated while keeping other ones down-regulated. In fact, the Uniprot keywords associated to STAT5A in Hipathia are *Lactation, Transcription*, and *Transcription regulation*, being all of them commonly significant to the three cancers, but presenting also a different behaviour: while the former is unidirectionally down-activated, the two latter ones are up-activated in KIRC, see Fig. 2c.

### Common functional profile in BRCA, KIRC and LUAD cancers

**Fig. 3:**
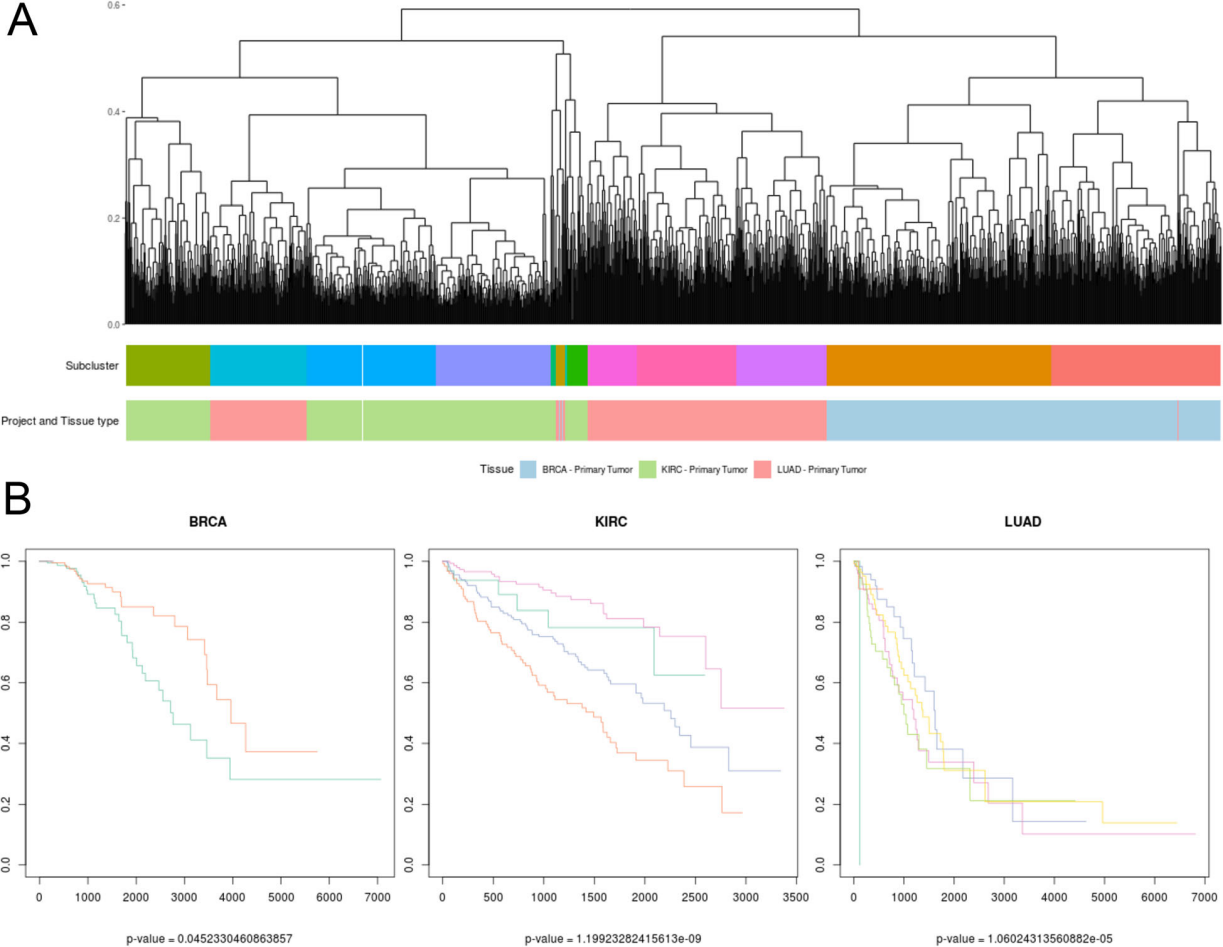
Survival analysis of cancer subtypes resulting from clustering by path activation values. **a**: Clustering of the tumor samples from BRCA, KIRC and LUAD based on the values of the paths which resulted significant in the comparison between healthy and tumor tissues in all three cancer types, colored by their tissue of origin (Tissue) and the subcluster in which they have been stratified. **b**: Kaplan-Meier curves of the subgroups created in each cancer, with the *p*-value of the survival analysis performed at the bottom. Curve colors are not matched with the subcluster colors but defined to be easily differentiated

The clear pattern shown in Fig. 3 is also distinguishable in the heatmaps of the common functional activities, both GO terms and Uniprot keywords, proving a remarkable ability of the functional data to discern between cancer and healthy groups, see Supplementary Figure SF4. After prioritizing the GO functions by the sum of the negative logarithm of the *p*-values in the comparisons of tumor vs. tissue samples for the three cancers, a list of unidirectionally down-activated common functions related with ion and water homeostasis and transport, and response to hormones emerged in the top 30 functions. Ions develop important roles in cancer progression, and deregulation levels may promote changes in the expression levels of ion channeling proteins, which can be related to identify different kinds of cancer and their severity [28].

We also found among the top 30 functions unidirectionally up-activated GO terms as *mRNA stabilization*, *Protein localization to kinetochore* and *Positive regulation of interferon-beta production* and bidirectional common functions up-activated in KIRC which include *Plasma membrane long-chain fatty acid transport, cGMP-mediated signaling*, *Nitric oxide mediated signal transduction* and *Positive regulation of receptor biosynthetic process.*

Other common results of our analysis included GO terms usually related to cancer, such as the histones H3 and H4 methylation and acetylation, DNA replication and recombination. Common up-activated GO functions, such as the regulation of adaptive immune response, the leukocyte migration, and the ones related to T cell activation and B cells apoptosis, hint at the complex relation between the immune system and the tumor microenvironment [29], highlighting tumor immune suppression. Also, we can observe an up-activated JAK-STAT cascade, strongly related to cell survival, migration and proliferation, making this signaling pathway an important indicator of tumorigenesis and, by definition, an important indicator of invasion, metastasis, proliferative state and tumor growth.

Regarding the common Uniprot keywords, after prioritizing the functions as above, we found a high number of ion transport related functions among the top 30 functions in the table, all of them unidirectionally down-activated. We also found *Chromosome partition* and *DNA replication* functions unidirectionally up-activated. With respect to the bidirectional top functions, we found *MHC I* and *MHC II*, which are down-activated in LUAD, and *Phagocytosis, Fatty acid metabolism* and *Fatty acid biosynthesis,* which are up-activated in KIRC. Interestingly, the latter two functions are regulated by path *PPAR signaling pathway: CD36*, which appeared in the top 10 significant paths in Table 3 also as up-activated in KIRC and down-regulated in LUAD and BRCA.

### Common features define survival related subgroups

After the selection of the common paths, a hierarchical clusterization was performed on the matrix of common path values for the tumor samples. As expected, due to the different tissue of origin, the clustering separated well among the different cancer types, specially BRCA from KIRC and LUAD, see Fig. 3a. Then, the tumor samples were divided into 13 groups (subclusters), according to the clusterization. These subclusters could be related to molecular subtypes for each cancer, grade or cancer progression stage in patients.

Guided by these results, we performed a survival analysis comparing the survival time of the individuals in the different groups of each cancer. Groups with less than 10 individuals of a cancer were filtered. Notice that each cancer includes samples from a different number of groups, for instance, BRCA samples are present in just two of the subgroups, while KIRC samples are present in four of the groups.

Interestingly, the survival time of the individuals in the groups resulted significantly different in all three cancers (*p-values* are 0.045 for BRCA, 1.199e-10 for KIRC and 1.060e-5 for LUAD, see Fig. 3b, note that the color code of the survival curves not correspond to color code of the subgroups). Similar results can be found for functional features (GO terms defined subgroups with *p*-values 0.065 for BRCA (filtering groups with less than 25 samples), 0 for KIRC and 0.001 for LUAD, Uniprot keywords defined subgroups with *p*-values 0.889 for BRCA, 0 for KIRC and 0.052 for LUAD), see Supplementary Figures SF5 and SF6.

### Tissue and cancer type prediction based on the common pathways

To further assess the sorting power of the common significant paths, these results were used as input for machine learning classification models. Two approaches of classification were analysed: the classification between Tumor and Normal Tissue in each one of the three cancers and the correct classification of Tumor tissue by cancer type.

The goal of these analyses is not to tune a machine learning algorithm to get the most predictive classification model from our data, but to check if our features are good enough to get significant results from easy-implemented, classical machine learning classification algorithms.

Through each one of these approaches we followed a conventional machine learning analysis pipeline: first, we explore the data to find the most suitable classification algorithm for our model based on preliminary classification metrics (such as accuracy and precision). After choosing the model, we deploy a full-fledged version based on the selected algorithm to assess said metrics and check out its usefulness as a classificator.

### Tumor tissue prediction

As input for tissue prediction, we provided the values of the common significant paths across the three types of cancer, making a specific dataset with Tumor and Normal Tissue labels for each one of them. To balance the number of Tumor and Normal Tissue samples, only the paired samples of each type of cancer were selected. To assess the predictive potential of the dataset, we also conducted two separate experiments based on the number of features: one input with 828 common paths and other with the top ten common paths found in Table 3.

As shown in Supplementary Table 4, the model comparison in each three cancers cast promising results across most of the algorithms, with accuracy values over 0.95, both with the full number of features and with the top ten paths. Based on these results, the model selected for this classification was K-Nearest-Neighbors (KNN) [30], which, again, provided excellent classification results in both data scenarios.

### Cancer type prediction

As in the previous case, the full number of common paths was compared with the top ten most significant ones. In this case, the dataset was composed by Tumor samples of each type of cancer. As we can see in Supplementary Table 5, the metrics seem better when we use all the significant paths, but it is interesting to note that, through KNN, we achieve an accuracy value over 90% with the top ten paths, which serves as an example of their relevancy as classificators and their potential to further develop artificial intelligence techniques.

### Survival related features

For each analyzed feature, samples were divided into three groups: the 20% of most activated samples, the 20% of lowest activated samples and the 60% of remaining samples, and the survival time of the different groups was compared. We found a number of pathways and functions related to survival depicted in Table 4. Surprisingly, the number of significant features is clearly unbalanced between KIRC and the other two cancers. Complete results of these analyses can be found in Supplementary Tables S6, S7 and S8.

**Table 4:** Number of significant survival-related features per cancer

	Paths	Gene Ontology	Uniprot
**BRCA**	14	0	2
**KIRC**	953	894	96
**LUAD**	29	10	1

Unfortunately, we found no common survival-related features significant in all three cancers at the same time. However, a number of survival-related features common to two of the three cancers were found: 31 paths, 6 Gene Ontology functions and 3 Uniprot keywords. Supplementary Figure SF6B shows the number of survival-related paths shared by each pair of cancer types.

Among the pairwise common survival-related paths we find the path *AMPK signaling pathway: CCNA2*, which was commonly up-activated along the three cancers (see Section *Common features* and Supplementary Figure SF3). This path has been significantly related to survival in KIRC and LUAD. In both cancers, a higher activity of this pathway is related to a poorer outcome, and a lower activity of the pathway is linked to a better outcome. Supplementary Figure SF6C shows the Kaplan-Meier curves for the three groups in KIRC (top) and LUAD (bottom).

### miRNA analysis

Finally and given that the pattern of miRNA expression can be correlated with cancer type, stage, and other clinical variables due their role as regulators of gene expression, we performed a miRNA analysis. We explored if the results at miRNA-level correspond with the ones found at pathway-level and made an analysis of the miRNA data provided by CAMDA. The main objective of this analysis was to obtain a list of the commonly significant miRNA, check their functions in a cancer context and relate them to the common significant paths.

The analysis consisted, as above, in the comparison of tumor vs. normal tissues across the three cancers separately and the selection of the commonly significant miRNAs, obtaining 229 common miRNAs as a result, 97 classified as unidirectionally upregulated, 30 as unidirectionally downregulated, and 102 as bidirectional. Table 5 shows the top ten most significant miRNA in all three cancers, after ordering them by the sum of the negative logarithm of the three *p-values*, together with their change in each cancer (UP or DOWN) and the function and cancer risk factor associated with their target gene based on previous literature. Most of them are closely related with cancer as oncogenes or tumor suppressors. The literature references their role in different types of cancer hallmarks [48, 49] such as cell proliferation and invasion. Some of them are also identified as prognostic markers in the three types of cancer, displaying, like in the case of mir-210 and mir-584, a bidirectional behaviour. Complete results of this analysis can be found in Supplementary Table S9.

**Table 5:** Top ten most significant miRNA, their cancer-type sign and prognosis factor according to the literature

Significant miRNA	BRCA	KIRC	LUAD	Function	Prognosis Factor
mir-21	UP	UP	UP	Oncogene, Targets tumor inhibitor proteins (LZTFL1)	Promotes proliferation and metastases [31, 32], UP: related to poor prognosis
mir-96	UP	DOWN	UP	Oncogene, MEK/ERK signaling by targeting AKR (BRCA)	Invasion, metastasis progression [33, 34], UP: poor prognosis
mir-139	DOWN	DOWN	DOWN	Tumor suppressor	Suppress proliferation, tumor growth and metastasis [35, 36], UP:better prognosis
mir-141	UP	DOWN	UP	Oncogene	Promotes proliferation and inhibits tumor cell apoptosis [37, 38], UP: poor prognosis
mir-183	UP	DOWN	UP	Oncogene	Cell viability, proliferation, invasion and metastasis [33, 39], UP: poor prognosis
mir-184	UP	DOWN	DOWN	Suppressor gene	Proliferation, invasion, apoptosis, [40] UP: better prognosis (BRCA), DOWN: better prognosis (KIRC)
mir-200	DOWN	DOWN	DOWN	Suppressor gene, TRAIL pathway, VEGF and VEGFR signaling network and epithelial-mesenchymal transition	Cancer invasion and metastasis, Level variation may correlate with disease progression [41], DOWN: better prognosis
mir-206	DOWN	DOWN	DOWN	Oncogene (BRCA), Tumor suppressor (LUAD, KIRC)	Cell invasion, migration, proliferation, Prognosis depend on cancer type [42–44]
mir-210	UP	UP	UP	Oncogene (BRCA, LUAD), Suppressor gene (KIRC)	Treatment resistance (tamoxifen) [45] UP: poor prognosis (BRCA, LUAD) and good prognosis (KIRC)
mir-584	UP	DOWN	DOWN	Tumor suppressor	UP: better prognosis (BRCA) DOWN: worse prognosis (KIRC) and better prognosis (LUAD) [46, 47]

To relate the miRNA activity with the common significant paths, we targeted the genes which each miRNA regulates and their presence in each one of the pathways studied. After this, we checked how many of these significant miRNAs were related with the common paths. We found 40 miRNAs (including mir-184 and mir-206, which were among the top 10 common miRNAs) targeting 787 of the 828 common paths. In particular, mir-206 shows relation with two of the top 10 common significant paths in Table 1: *Melanogenesis: TYRP1* and *Aldosterone-regulated sodium reabsorption: KCNJ1.* The complete relation of miRNAs and their targeting nodes and paths can be found in Supplementary Table S10.

## Discussion

In this work we presented our approach to the CAMDA 2019 Hi-Res Cancer Data Integration Challenge. Our goal was to find common functional patterns in the three cancers at a pathway and functional level, for which we used the pathway analysis tool Hipathia. One of the advantages of using Hipathia with respect to other classical pathway and functional enrichment tools is the fact that path activity values, as well as functional activity values, are computed for each of the samples in the study, allowing a set of a posteriori analysis based on these features, such as the clustering or survival stratification applied in this work. With traditional functional enrichment methods it is not possible to classify the samples by their functional activity, since we don’t have information for individual samples, but just the enrichment of the tested comparison.

On the other hand, some of the main drawbacks of the method are the fact that it takes the gene expression level as a proxy of the protein level, and that it does not take into account post-transcriptional modifications which may appear downstream and are specially relevant in cancer [50]. However, Hipathia has shown that it is both highly sensitive and specific, outperforming many other pathway analysis tools [51].

Based on the results obtained at pathway level, we find that most of the significant findings are closely related to hormone-based signaling pathways and ion channels and homeostasis. Ion channel dysfunctions have an important contribution to cancer hallmarks that is increasingly becoming elucidated. Several ions can work as signaling molecules (second messengers) for different cellular processes such as cell cycle control, apoptosis and migration [52, 53]. Regarding hormones, about two-thirds of all BRCAs diagnosed are hormone dependent indicating the crucial role they play in BRCA progression as observed in hormone dependent BRCA molecular subtypes as luminal A or luminal B [54, 55]. The influence of hormones such as melatonin or growth hormone has been reported in KIRC cancer [56, 57] and thyroid hormone in LUAD [58].

Remarkably, we were also able to identify alternative activation of cancer-specific paths as shown in STAT5 as exemple. Alternative activation paths are gaining relevance as a potential source of cancer-specific biomarkers as described in [59–61]. From the clinical point of view, this knowledge could be the key to design novel strategies and treatments for specific cancer types for which our findings could be used as the basis for further research.

Along with easily interpretable results, some significant paths and functions that seem to be unrelated to cancer also appeared, such as the *Spermatogenesis pathway* or the function *Lactation*. However, a closer look to these results shows that most of these paths share a relevant number of genes with tumor related pathways, and most of the functions are annotated to genes with tumoral effect. Such is the case of *Spermatogenesis,* which is closely related to MAPK, AMPK and TGF-β signaling pathways, of special relevance in tumorigenesis [62], and function *Lactation,* which is annotated to STAT5A, an important element in the development of a wide array of cancers [63] but also a critical part in inducing lactation in women through the ErbB pathway.

Common significant paths have proven to be also useful to further classify cancer types into subgroups with a significant difference in their survival time. These findings deserve further study to discern whether the grouping identifies different molecular subtypes or is driven by cancer stages, but they point out the potential of the selected paths as prognosis markers. Going one step further to prove their prognostic potential, we also proved that the common significant paths could be used as inputs for machine learning models, since they return high accuracy results in the classification of tumor versus normal samples, as well as in the classification of the three types of cancer. To check the validity of the results, a significant effort has been realized to eliminate possible bias related to the used model, as overfitting [64]. The use of alternative metrics, such as the area under the ROC curve could be useful to further assess the possibilities of this dataset as machine learning input.

After the selection of the significant features in the survival analysis, it stands out an unbalanced higher number of features related to survival in KIRC. The reasons for such results would require a full new specific analysis centered in the KIRC cancer which is out of the scope of the current work, but which would constitute an interesting starting point for further research.

Regarding the results found in the miRNA analysis, the large number of genes and, therefore, paths related to the significant miRNAs are not surprising: a single miRNA has multiple binding sites and can target a vast array of genes at post-transcriptional level [65]. Nonetheless, specific and meaningful relations can be traced between significant miRNAs and the genes that comprise the studied pathways. For instance, mir-184 binds to the BLC2 gene, which is part of multiple pathways, such as *Apoptosis* and *Ras*, that are closely related to cancer, specially in LUAD and KIRC [66].

## Conclusions

Despite clinical advances, cancer research through omics data still remains a challenging field of work. Thanks to initiatives such as the CAMDA challenges, the gap between omics sciences and clinical research closes, making relevant datasets available for the scientific community to freely explore and test with novel methodologies.

In the context of the CAMDA 2019 Hi-Res Cancer Data Integration Challenge, we analyzed pathway and functional activity of breast, kidney and lung cancers to identify underlying common patterns across them. We found 828 subpathways, 912 Gene Ontology terms and 91 Uniprot keywords commonly significant to the three studied tumors. Such features show the power to classify tumor samples into subgroups with different survival times, and predict tumor state and tissue of origin through machine learning techniques. We also identified alternative activation of pathways based on cancer type, which represents a fine tuning of traditional pathway approaches and could lead to therapeutic applications.

## Material and methods

### Data download and normalization

Data provided for the CAMDA 2019 Hi-Res Cancer Data Integration Challenge consisting of RNA-Seq and miRNA raw counts matrices were downloaded from the SFTP server hosted at BOKU Vienna. Data came from 3 different cancer types: breast cancer (TCGA-BRCA project, with 656 samples, corresponding to 589 Primary Tumor samples and 67 Solid Tissue Normal samples), kidney cancer (TCGA-KIRC project, with 602 samples, corresponding to 530 Primary Tumor samples and 72 Solid Tissue Normal samples) and lung adenocarcinoma (TCGA-LUAD project, with 574 samples, corresponding to 515 Primary Tumor samples and 59 Solid Tissue Normal samples). RNA-Seq and miRNA data was subsequently normalized with TMM normalization [67] and log transformed separately, creating six different data matrices (one per each omics and cancer type). Survival data was downloaded from the GDC data portal [10].

### Pathway & functional level computation

The matrix of normalized gene expression was scaled between 0 and 1, and transformed to a matrix of subpathway activation values by means of the *Hipathia* Bioconductor package [11]. In Hipathia, pathways are divided into *subpathways*, which represent the path linking any input node with a final effector protein in the graph, and are therefore also referred to as *paths*. Paths are identified by a name including the pathway from which they come from, and the name of the final effector protein to which signal arrives. Asterisks are used to differentiate paths when final effector proteins are not unique in a pathway. As an example, *ErbB signaling pathway: STAT5A* and ErbB signaling pathway: STAT5A* represent two different paths in the ErbB signaling pathway ending in two different instances of node STAT5A, see Fig. 2b. The methodology computes a value representing the activity of each of the analyzed paths from the gene expression data by means of a two-step algorithm: firstly, an expression score is computed for each node based on their containing genes, and secondly, the value of the signal passing through the path until the last node is inferred depending on the topology of the path. Hipathia uses the pathway information from the Kyoto Encyclopedia of Genes and Genomes (KEGG) [68] as layout for the topology of the pathways. The procedure is applied to each analyzed path for each sample separately, transforming the matrix of gene expression values into a matrix of path activity values. From this new matrix, the matrices of functional activities corresponding to Gene Ontology functions and Uniprot keywords were computed with *Hipathia*, taking into account the different pathways related to each one of the functions. A total of 1868 paths (from 146 KEGG signaling pathways), 1654 Gene Ontology functions and 142 Uniprot Keywords were analyzed.

### Tumor vs. normal tissue comparisons

For each cancer type, a comparison between the gene, miRNA, path and functional activation levels of the *Solid Tissue Normal* and *Primary Tumor* classes was performed with the *lmFit* and *eBayes* functions from the *limma* package [69]. The FDR [70] correction method was used to adjust the multiple testing effects on the *p-values*, and a cutoff of alpha = 0.05 was established to determine statistical significance. Heatmaps with the features with a significant *p-value* and an absolute value of the logarithm of the Fold Change (logFC) greater than 0.3 were plotted, allowing a non-supervised clustering method (*hclust* function, “complete” method) to establish the order for the rows and columns by similarity. An UpSet plot [71] representing the intersections of the significant paths or functions in each cancer was created with package *UpSetR* [72].

### Common features

Common results at a path, function and miRNA level were established by selecting the features with statistical significance in the tumor vs. normal tissue comparisons in all three cancers. Features presenting also a common sign of the logFC in all three cancers were categorized as unidirectional (changes are always in the same direction: up or down activated), and those with a different sign of the logFC as bidirectional. Since we analyzed three cancers, common bidirectional features always include two cancers with the same logFC and one cancer which differs from them. Therefore, we can identify these groups by the sign and cancer from the one which differs. As an example, KIRC UP will denote the group of features which are up-activated in KIRC but down-activated in both BRCA and LUAD.

### Subtype classification

The matrices of the path, GO terms and Uniprot keywords values were filtered by the features selected as common as described above, and subsequently normalized by rows between 0 and 1. Each matrix was clustered using the *hclust* function and 1 - the correlation between samples divided by 2 as distance. The resulting clustering was cut with *cutree* to create subclusters. The survival of the donors in the groups resulting from this partition were analyzed with the function *survdiff* from the *survival* R package [73], which returns a Chi-squared statistic which is used to calculate a *p-value*. A significance cutoff of 0.05 is established. Kaplan - Meier curves [74] were plotted to visualize survival differences among the defined groups.

### Tissue and subtype prediction

A standard machine learning pipeline was followed in order to analyze the predictive capability of the data under a classification model. This pipeline comprises the following three steps: Exploratory Data Analysis (EDA), classification algorithm selection and model building and testing. The EDA was done through R to explore value distribution and correlation among variables, proportion of samples belonging to each analysed group and group-based sample distribution related to Principal Component Analysis (PCA) results [75] using R packages *ellipse*, *ggplot2* [76], *mixOmics* [77], *reshape2* [78] and *RcolorBrewer*. For the selection of a suiting classification algorithm, Python packages *pandas* [79], *numpy*, [80], *matplotlib* [81] and *sci-kit learn* [82] were imported. The process of selection was based on using the previously scaled variable matrix (X) and the vector with each sample corresponding group label (y) as a basis for a K-fold cross validation [83] involving an assortment of pre-selected classification algorithms. The final verdict comes from averaging the resulting accuracy of each model and assessing its standard deviation: the models with higher accuracy and lower standard deviation will be favoured. The models used were provided by *sci-kit learn* package, comprising classification algorithms of varying complexity and able to work in scenarios were there are more than two classification groups: Decision Tree Classifiers [84], Gaussian Naive Bayes [85], K-Nearest-Neighbors Classifier [30], K-Means Clustering [86] and Random Forest Classifier [87]. Finally, once an algorithm is selected, a standalone model is made, using again *sci-kit learn* Python package. To assess this model utility, we approach a classical data split into train, test and validation, with a distribution of 60% data train, 20% to test and 20% to validation.

### Survival-related pathways and functions

For each analyzed feature, samples were divided into three groups: 20% of most activated samples, 20% of lowest activated samples and the 60% of remaining samples. An analysis including function *survdiff* from the *survival* R package [73] was performed on each feature, which returns a Chi-squared statistic which is used to calculate a *p-value*. The FDR method [70] is used as above to correct for multiple testing effects. Kaplan - Meier curves [74] were plotted to visualize survival differences among the defined groups. Pairwise common survival-related features were established by selecting those with a significant *p-value* in two different cancers at the same time. UpSet plots [71] representing the number of overlapping survival-related pathways or functions were created with package *UpSetR* [72].

## Supplementary Information

**Additional file 1: Figure SF1.** Graphical analysis of the common GO terms across the three cancers. Description: A: Upset plot representing the number of coincident significant GO terms between cancers. For each cancer type, two groups have been created: the group of the up-activated GO terms (denoted by UP), and the group of the down-activated GO terms (denoted by DOWN). Therefore, the same GO can not be at the same time in the same cancer’s UP and DOWN groups. Red and blue horizontal bars represent the number of significant GOs in each group. Each vertical bar in the plot represents the intersection of the groups in the inferior rows with a solid point, and the exclusion of the groups in the inferior rows with a shaded point. An orange box surrounds the part of the UpSet plot representing the GO terms which are significant in all three cancers. The blue and red vertical bars represent the GOs which are simultaneously down- and up-regulated in the three cancers, respectively. B: Heatmaps of the significantly common GO terms values, represented inside the orange box of the UpSet plot above. Samples and rows were ordered following the results of a hierarchical clusterization. Tumor samples are colored in blue while normal tissue samples are colored in light blue. In the heatmap, higher activation values are colored in red and lower activation values in blue. Left: BRCA cancer data. Center: KIRC cancer data. Right: LUAD cancer data.

**Additional file 2: Figure SF2**. Graphical analysis of the common Uniprot functions across the three cancers. Description: A: Upset plot representing the number of coincident significant Uniprot Keywords between cancers. For each cancer type, two groups have been created: the group of the up-activated Uniprot Keywords (denoted by UP), and the group of the down-activated Uniprot Keywords (denoted by DOWN). Therefore, the same GO can not be at the same time in the same cancer’s UP and DOWN groups. Red and blue horizontal bars represent the number of significant GOs in each group. Each vertical bar in the plot represents the intersection of the groups in the inferior rows with a solid point, and the exclusion of the groups in the inferior rows with a shaded point. An orange box surrounds the part of the UpSet plot representing the Uniprot Keywords which are significant in all three cancers. The blue and red vertical bars represent the GOs which are simultaneously down- and up-regulated in the three cancers, respectively. B: Heatmaps of the significantly common Uniprot Keywords values, represented inside the orange box of the UpSet plot above. Samples and rows were ordered following the results of a hierarchical clusterization. Tumor samples are colored in blue while normal tissue samples are colored in light blue. In the heatmap, higher activation values are colored in red and lower activation values in blue. Left: BRCA cancer data. Center: KIRC cancer data. Right: LUAD cancer data.

**Additional file 3: Figure SF3**. Alternative path activation related to *AMPK signaling pathway: CCNA2.* Description: A) Boxplots representing the distribution of the activity values for the *AMPK signaling pathway: CCNA2* path (top) and the Uniprot keyword Mitosis (bottom). Expression values are grouped by tissue type (tumor or normal) and cancer. B) Up and down regulation of genes in the *AMPK signaling pathway: CCNA2* path in BRCA (top), LUAD (center) and KIRC (bottom). Blue nodes correspond to significant down-regulated genes, red nodes correspond to significant up-regulated genes and white nodes correspond to non-significant nodes. Red lines are depicted because the whole activity of the pathway is significantly up-activated after a statistical analysis.

**Additional file 4: Figure SF4**. Heatmaps of function activation for the three cancers. Description: Heatmaps of function activations for the three cancers. Samples and functions were ordered following the results of a non-supervised hierarchical clusterization. Top row corresponds to Gene Ontology functions and bottom row to Uniprot keywords. Each column represents a cancer, from left to right: breast, kidney and lung cancers.

**Additional file 5: Figure SF5**. Survival analysis of cancer subtypes resulting from clustering by GO terms values. Description: A: Clustering of the tumor samples from BRCA, KIRC and LUAD based on the values of the GO terms which resulted significant in the comparison between healthy and tumor tissues in all three cancer types, colored by their tissue of origin (Tissue) and the subcluster in which they have been stratified. B: Kaplan-Meier curves of the subgroups created in each cancer, with the *p*-value of the survival analysis performed at the bottom. Curve colors are not matched with the subcluster colors but defined to be easily differentiated. **Figure SF6**. Survival analysis of cancer subtypes resulting from clustering by Uniprot function values. Description: A: Clustering of the tumor samples from BRCA, KIRC and LUAD based on the values of the Uniprot keywords which resulted significant in the comparison between healthy and tumor tissues in all three cancer types, colored by their tissue of origin (Tissue) and the subcluster in which they have been stratified. B: Kaplan-Meier curves of the subgroups created in each cancer, with the p-value of the survival analysis performed at the bottom. Curve colors are not matched with the subcluster colors but defined to be easily differentiated. **Figure SF7**. Specific functions per cancer and survival related to *AMPK signaling pathway: CCNA2.* Description: A) UpSet plot indicating the number of paths in the pairwise intersections among the three analyzed cancers, and the (null) intersection of the three of them. C) Kaplan-Meier curves for the three groups of activation intensity defined by path *AMPK signaling pathway: CCNA2* in KIRC (top) and LUAD (bottom). Blue lines correspond to the 20% of samples with lowest activity values, red lines correspond to the 20% of samples with highest activity values of this pathway and orange lines correspond to the remaining 60% of samples.

**Additional file 6: Table S1**. Results table for the comparison of normal versus tumor tissue of path values. Description: Rows represent the features analyzed. Columns: *feature* is the Hipathia identifier, *name* is the human readable name, *common* is a boolean indicating whether the feature is significant in all three cancers, *type* indicates whether the feature is unidirectional, bidirectional or not common, *subtype* classifies common features into 8 subgroups (unidirectional can be ALL UP or ALL DOWN, indicating whether the feature has positive or negative statistic in all three cancers, respectively, and bidirectional are represented by the cancer that differs from the other two, so that KIRC UP represent the group of features which a positive statistic in KIRC and negative one in BRCA and LUAD), *combined. PV* is the sum of the negative logarithms in base 10 of the comparison *p*-values in the three cancers, *sign.[cancer]* is the direction of the change in cancer [cancer], *stat.[cancer]* is the statistic of the comparison in cancer [cancer] and *adj. PV.[cancer]* is the FDR adjusted *p*-value of the comparison in cancer [cancer].

**Additional file 7: Table S2**. Results table for the comparison of normal versus tumor tissue of Gene Ontology (GO) term values. Description: Rows represent the features analyzed. Columns: *feature* is the GO identifier, *common* is a boolean indicating whether the feature is significant in all three cancers, *type* indicates whether the feature is unidirectional, bidirectional or not common, *subtype* classifies common features into 8 subgroups (unidirectional can be ALL UP or ALL DOWN, indicating whether the feature has positive or negative statistic in all three cancers, respectively, and bidirectional are represented by the cancer that differs from the other two, so that KIRC UP represent the group of features which a positive statistic in KIRC and negative one in BRCA and LUAD), *combined. PV* is the sum of the negative logarithms in base 10 of the comparison *p*-values in the three cancers, *sign.[cancer]* is the direction of the change in cancer [cancer], *stat.[cancer]* is the statistic of the comparison in cancer [cancer] and *adj. PV.[cancer]* is the FDR adjusted *p*-value of the comparison in cancer [cancer].

**Additional file 8: Table S3**. Results table for the comparison of normal versus tumor tissue of Uniprot keyword values. Description: Rows represent the features analyzed. Columns: *feature* is the Uniprot identifier, *common* is a boolean indicating whether the feature is significant in all three cancers, *type* indicates whether the feature is unidirectional, bidirectional or not common, *subtype* classifies common features into 8 subgroups (unidirectional can be ALL UP or ALL DOWN, indicating whether the feature has positive or negative statistic in all three cancers, respectively, and bidirectional are represented by the cancer that differs from the other two, so that KIRC UP represent the group of features which a positive statistic in KIRC and negative one in BRCA and LUAD), *combined. PV* is the sum of the negative logarithms in base 10 of the comparison p-values in the three cancers, *sign.[cancer]* is the direction of the change in cancer [cancer], *stat.[cancer]* is the statistic of the comparison in cancer [cancer] and *adj. PV.[cancer]* is the FDR adjusted p-value of the comparison in cancer [cancer].

**Additional file 9: Table S4**. Metrics results from the algorithm comparison through K-Fold Cross-Validation to classify tumor and healthy tissue. Description: The comparison has been made by cancer type (BRCA, LUAD, KIRC), taking in account either all significant paths or only the top ten most significant ones. The algorithms used were K-Nearest-Neighbor (KNN), Decision Tree (CART), Naive-Bayes Classificator (NB), K-Means clustering (KMN) and Random Forest (RF). The metrics analysed take account of the mean Accuracy of the model testing across all the possible training and test combinations data splits resulting from the K-Fold Cross-Validation, along with its associated Standard Deviation.

**Additional file 10: Table S5**. Metrics results from the algorithm comparison through K-Fold Cross-Validation to classify three types of tumors. Description: The comparison has been made by grouping the tumor samples of the three types of cancer (BRCA, LUAD, KIRC), taking in account either all significant paths or only the top ten most significant ones. The algorithms used were K-Nearest-Neighbor (KNN), Decision Tree (CART), Naive-Bayes Classificator (NB), K-Means clustering (KMN) and Random Forest (RF). The metrics analysed take account of the mean Accuracy of the model testing across all the possible training and test combinations data splits resulting from the K-Fold Cross-Validation, along with its associated Standard Deviation.

**Additional file 11: Table S6**. Results table for the survival analysis of path values. Description: Rows represent the features analyzed. Columns: *feature* is the Hipathia path identifier, *combined. PV* is the sum of the negative logarithms in base 10 of the survival *p*-values in the three cancers, *PV.[cancer]* is the raw *p*-value of the survival analysis in cancer [cancer] and *adj. PV.[cancer]* is the FDR adjusted *p*-value of the survival analysis in cancer [cancer].

**Additional file 12: Table S7**. Results table for the survival analysis of GO term values. Description: Rows represent the features analyzed. Columns: *feature* is the GO identifier, *combined. PV* is the sum of the negative logarithms in base 10 of the survival *p*-values in the three cancers, *PV.[cancer]* is the raw *p*-value of the survival analysis in cancer [cancer] and *adj. PV.[cancer]* is the FDR adjusted *p*-value of the survival analysis in cancer [cancer].

**Additional file 13: **Table S8. Results table for the survival analysis of Uniprot keyword values. Description: Rows represent the features analyzed. Columns: *feature* is the Uniprot identifier, *combined. PV* is the sum of the negative logarithms in base 10 of the survival *p*-values in the three cancers, *PV.[cancer]* is the raw *p*-value of the survival analysis in cancer [cancer] and *adj. PV.[cancer]* is the FDR adjusted *p*-value of the survival analysis in cancer [cancer].

**Additional file 14: Table S9**. esults table for the comparison of normal versus tumor tissue of miRNA values. Description: Rows represent the features analyzed. Columns: *feature* is the miRNA identifier, *common* is a boolean indicating whether the feature is significant in all three cancers, *type* indicates whether the feature is unidirectional, bidirectional or not common, *subtype* classifies common features into 8 subgroups (unidirectional can be ALL UP or ALL DOWN, indicating whether the feature has positive or negative statistic in all three cancers, respectively, and bidirectional are represented by the cancer that differs from the other two, so that KIRC UP represent the group of features which a positive statistic in KIRC and negative one in BRCA and LUAD), *combined. PV* is the sum of the negative logarithms in base 10 of the comparison *p*-values in the three cancers, *sign.[cancer]* is the direction of the change in cancer [cancer], *stat.[cancer]* is the statistic of the comparison in cancer [cancer] and *adj. PV.[cancer]* is the FDR adjusted *p*-value of the comparison in cancer [cancer].

**Additional file 15: Table S10**. Table of miRNA, gene and path relations. Description: Rows represent relations. Columns include: *miRNA*, the name of the miRNA, *gene*, the gene targeted by the miRNA, *node*, Hipathia code of the node including the gene, *node.label*, readable name of the node, *path*, Hipathia name of the path including the node, *path.label*, readable name of the path including the node, *pathway*, KEGG pathway code including the path, and *pathway.label*, name of the KEGG pathway including the path.

## Data Availability

The datasets analysed during the current study are available in the GDC Data Portal repository, *http://gdc.cancer.gov**.*
