# Supplementary figures and images for: Common pathways and functional profiles reveal underlying patterns in Breast, Kidney and Lung cancers
Source: Biol Direct. 2021 May 26;16:9. doi: 10.1186/s13062-021-00293-8 (PMC8152308; doi:10.1186/s13062-021-00293-8)

A

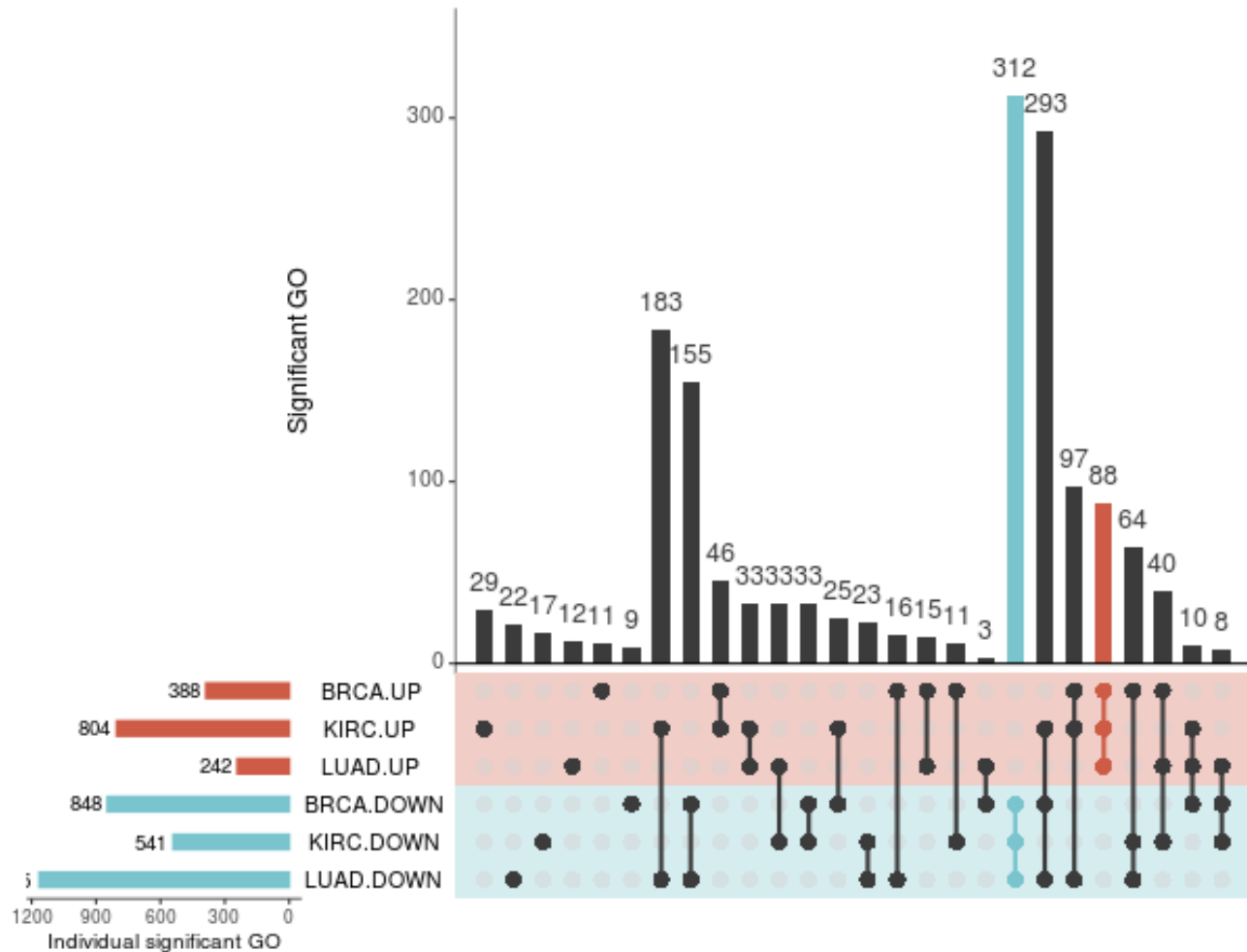

B

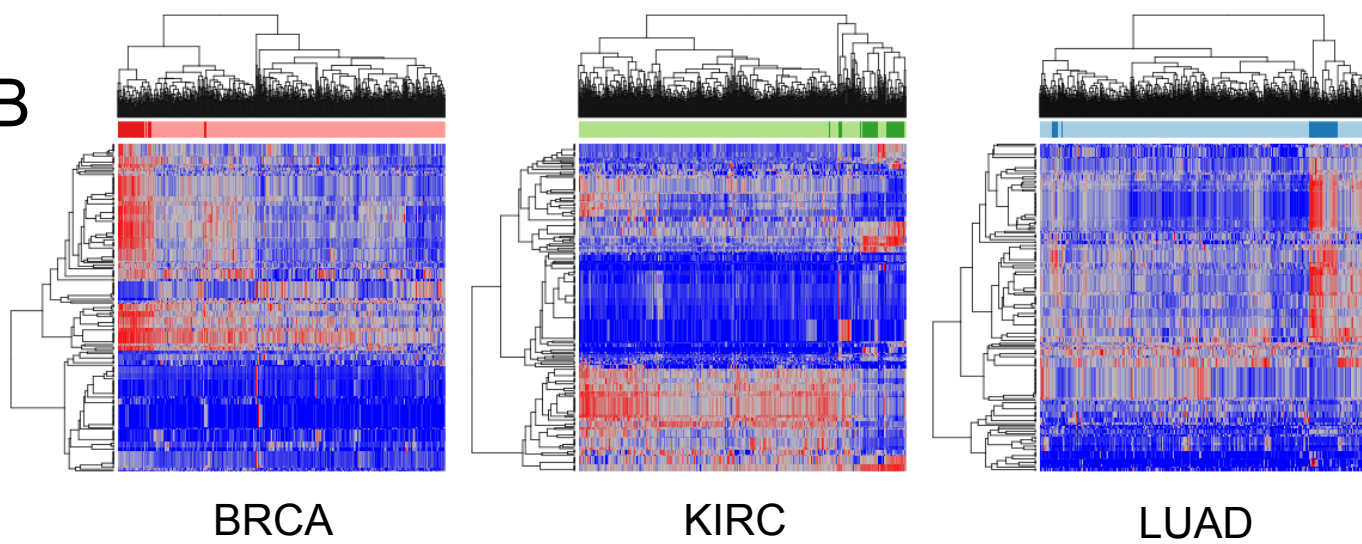

Supplement: Supplementary file 1 — Additional file 1: Figure SF1. Graphical analysis of the common GO terms across the three cancers. Description: A: Upset plot representing the number of coincident significant GO terms between cancers. For each cancer type, two groups have been created: the group of the up-activated GO terms (denoted by UP), and the group of the down-activated GO terms (denoted by DOWN). Therefore, the same GO can not be at the same time in the same cancer’s UP and DOWN groups. Red and blue horizontal bars represent the number of significant GOs in each group. Each vertical bar in the plot represents the intersection of the groups in the inferior rows with a solid point, and the exclusion of the groups in the inferior rows with a shaded point. An orange box surrounds the part of the UpSet plot representing the GO terms which are significant in all three cancers. The blue and red vertical bars represent the GOs which are simultaneously down- and up-regulated in the three cancers, respectively. B: Heatmaps of the significantly common GO terms values, represented inside the orange box of the UpSet plot above. Samples and rows were ordered following the results of a hierarchical clusterization. Tumor samples are colored in blue while normal tissue samples are colored in light blue. In the heatmap, higher activation values are colored in red and lower activation values in blue. Left: BRCA cancer data. Center: KIRC cancer data. Right: LUAD cancer data. [file 13062_2021_293_MOESM1_ESM.pdf]

A

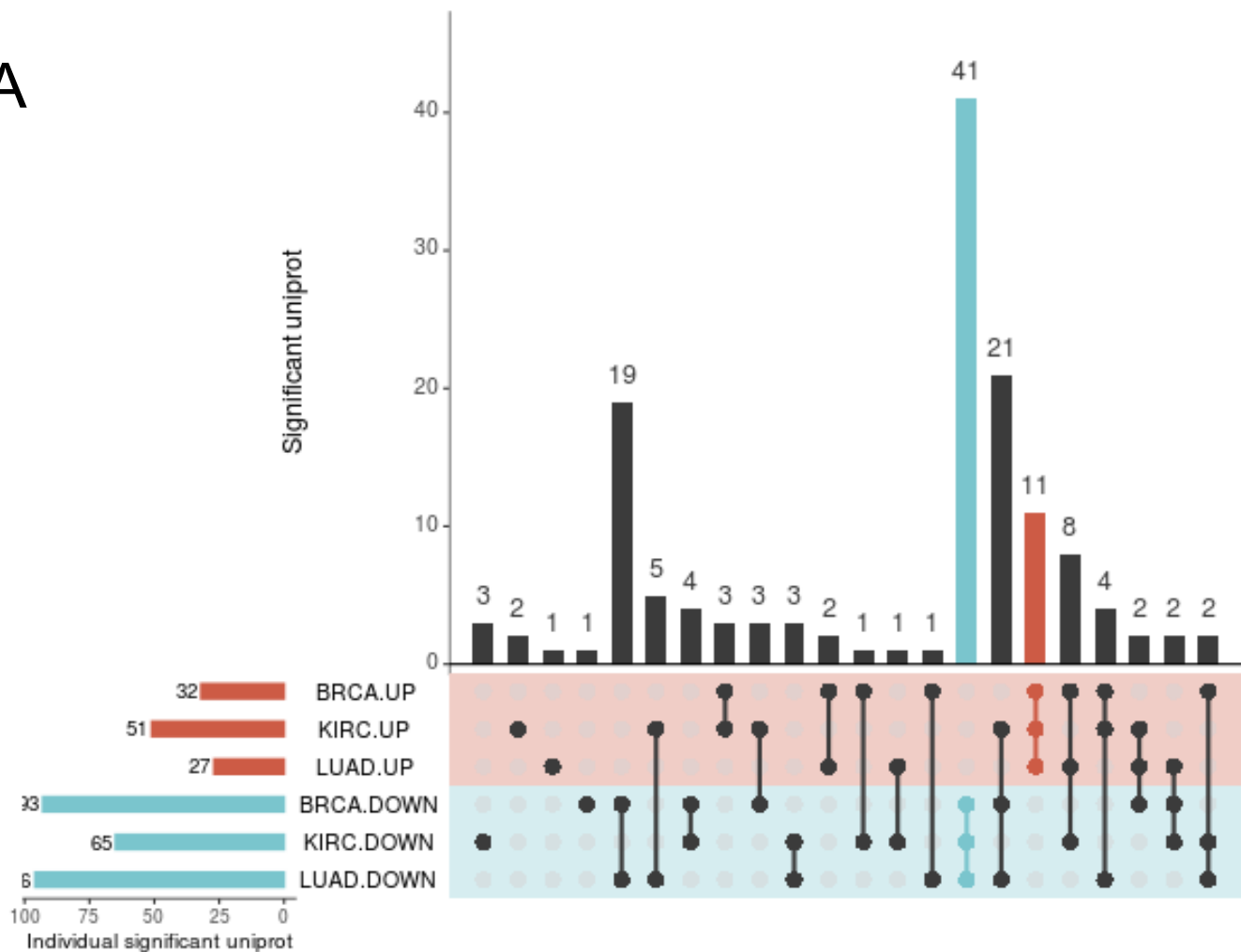

B

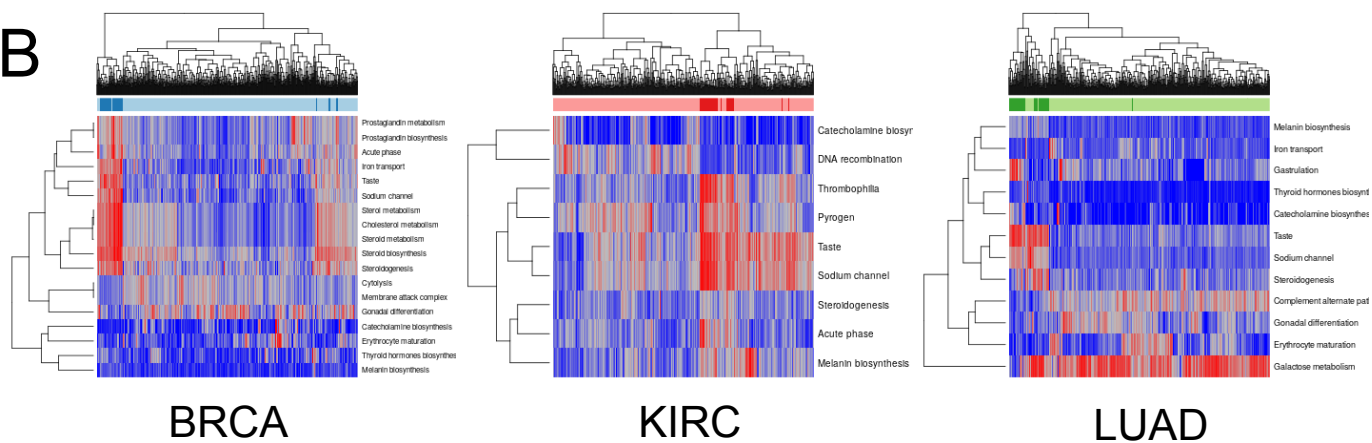

Supplement: Supplementary file 2 — Additional file 2: Figure SF2. Graphical analysis of the common Uniprot functions across the three cancers. Description: A: Upset plot representing the number of coincident significant Uniprot Keywords between cancers. For each cancer type, two groups have been created: the group of the up-activated Uniprot Keywords (denoted by UP), and the group of the down-activated Uniprot Keywords (denoted by DOWN). Therefore, the same GO can not be at the same time in the same cancer’s UP and DOWN groups. Red and blue horizontal bars represent the number of significant GOs in each group. Each vertical bar in the plot represents the intersection of the groups in the inferior rows with a solid point, and the exclusion of the groups in the inferior rows with a shaded point. An orange box surrounds the part of the UpSet plot representing the Uniprot Keywords which are significant in all three cancers. The blue and red vertical bars represent the GOs which are simultaneously down- and up-regulated in the three cancers, respectively. B: Heatmaps of the significantly common Uniprot Keywords values, represented inside the orange box of the UpSet plot above. Samples and rows were ordered following the results of a hierarchical clusterization. Tumor samples are colored in blue while normal tissue samples are colored in light blue. In the heatmap, higher activation values are colored in red and lower activation values in blue. Left: BRCA cancer data. Center: KIRC cancer data. Right: LUAD cancer data. [file 13062_2021_293_MOESM2_ESM.pdf]

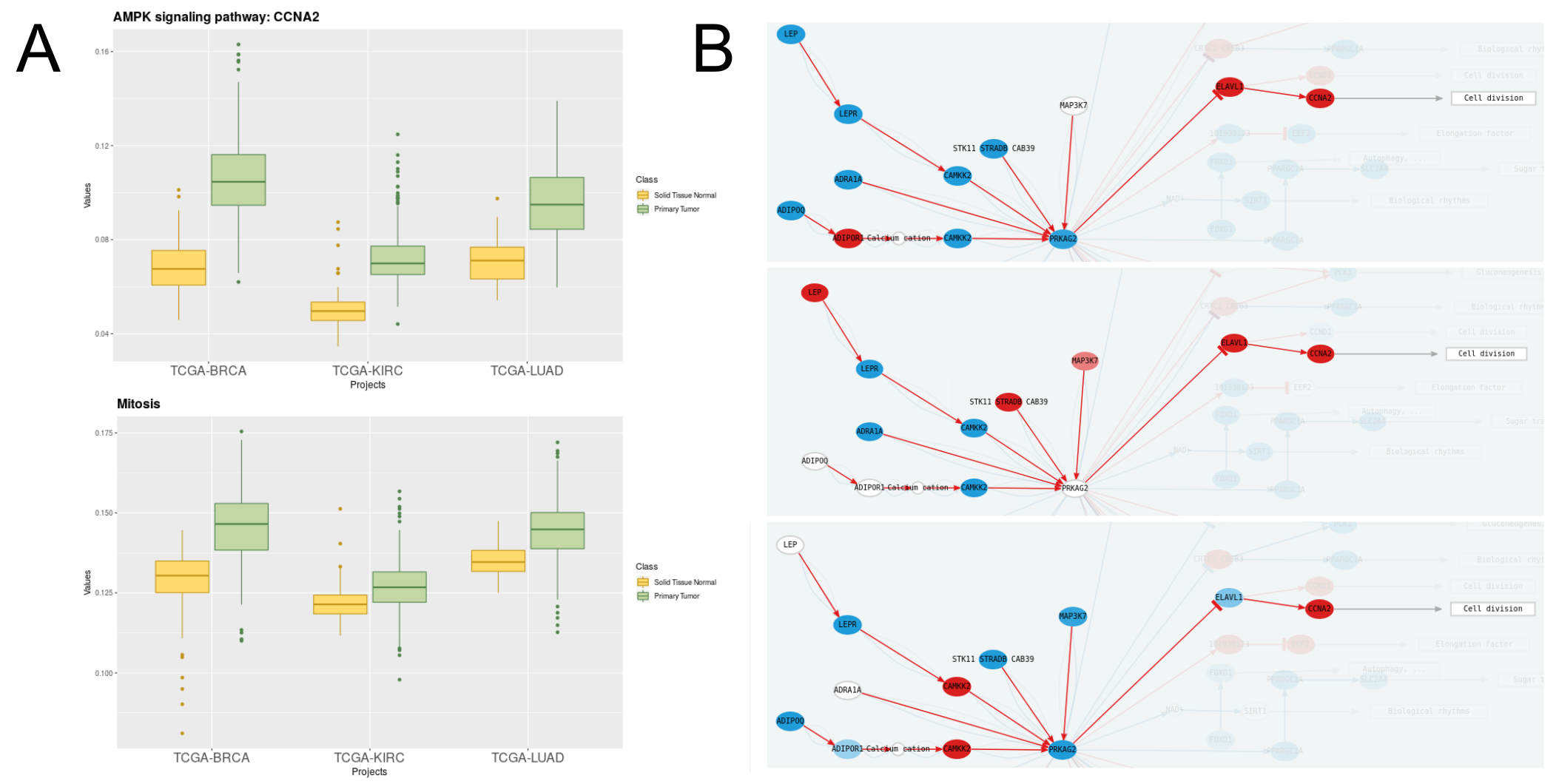

Supplement: Supplementary file 3 — Additional file 3: Figure SF3. Alternative path activation related to AMPK signaling pathway: CCNA2. Description: A) Boxplots representing the distribution of the activity values for the AMPK signaling pathway: CCNA2 path (top) and the Uniprot keyword Mitosis (bottom). Expression values are grouped by tissue type (tumor or normal) and cancer. B) Up and down regulation of genes in the AMPK signaling pathway: CCNA2 path in BRCA (top), LUAD (center) and KIRC (bottom). Blue nodes correspond to significant down-regulated genes, red nodes correspond to significant up-regulated genes and white nodes correspond to non-significant nodes. Red lines are depicted because the whole activity of the pathway is significantly up-activated after a statistical analysis. [file 13062_2021_293_MOESM3_ESM.png]

BRCA

KIRC

LUAD

GO  
terms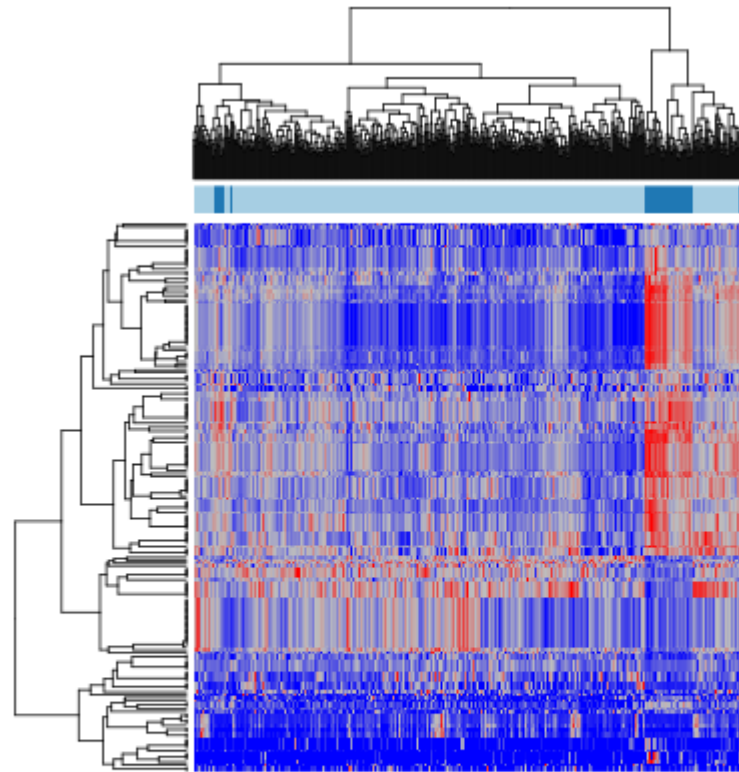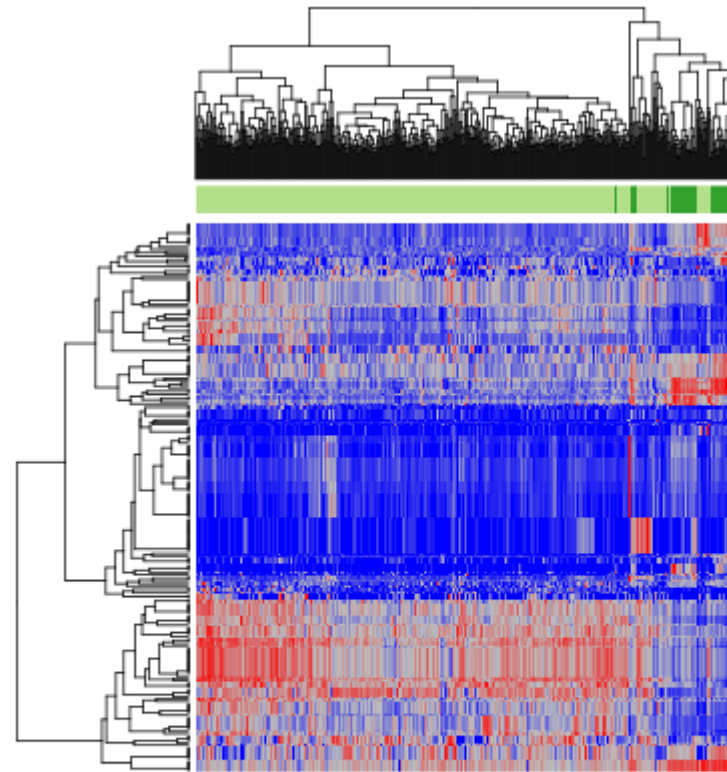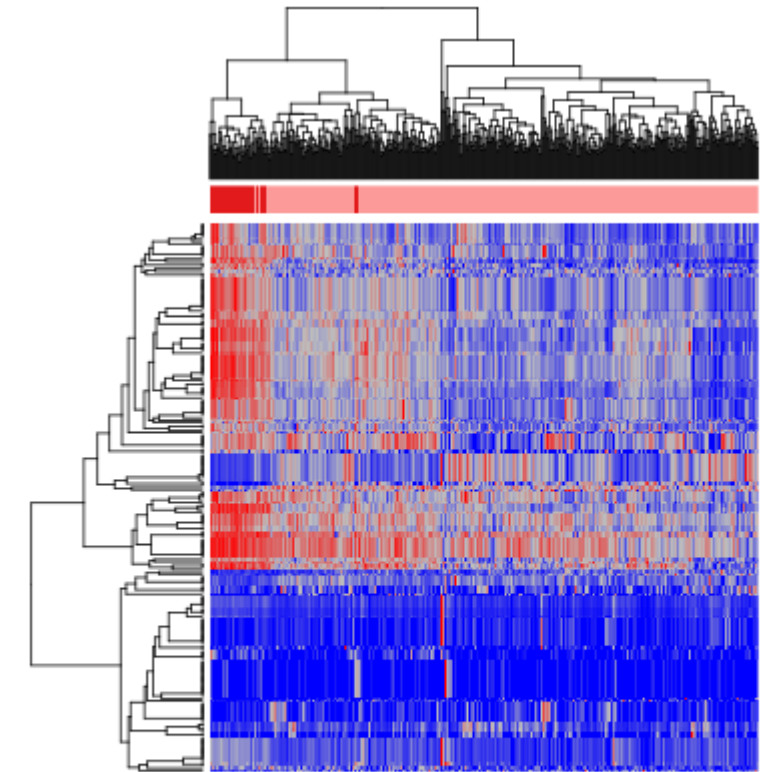Uniprot  
keywords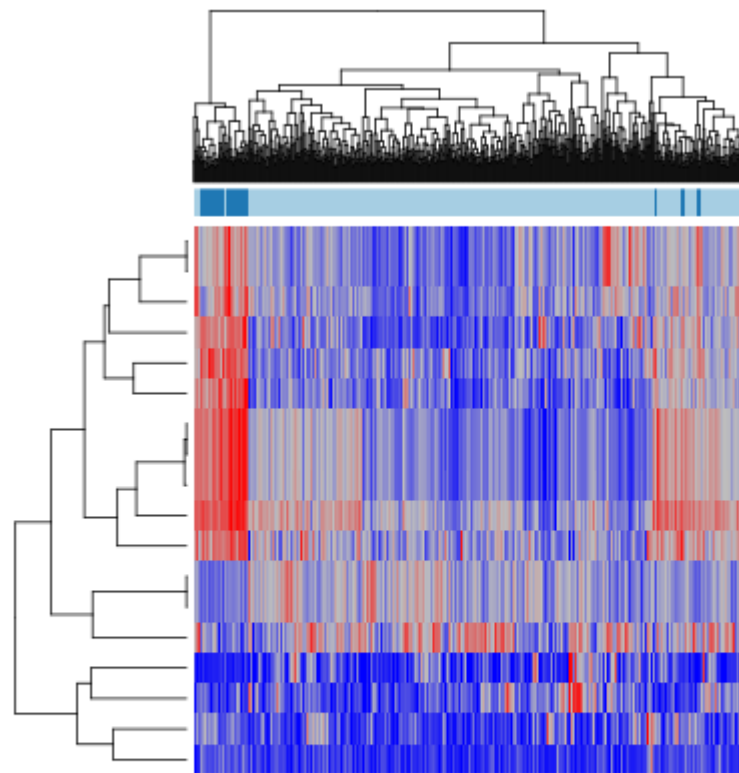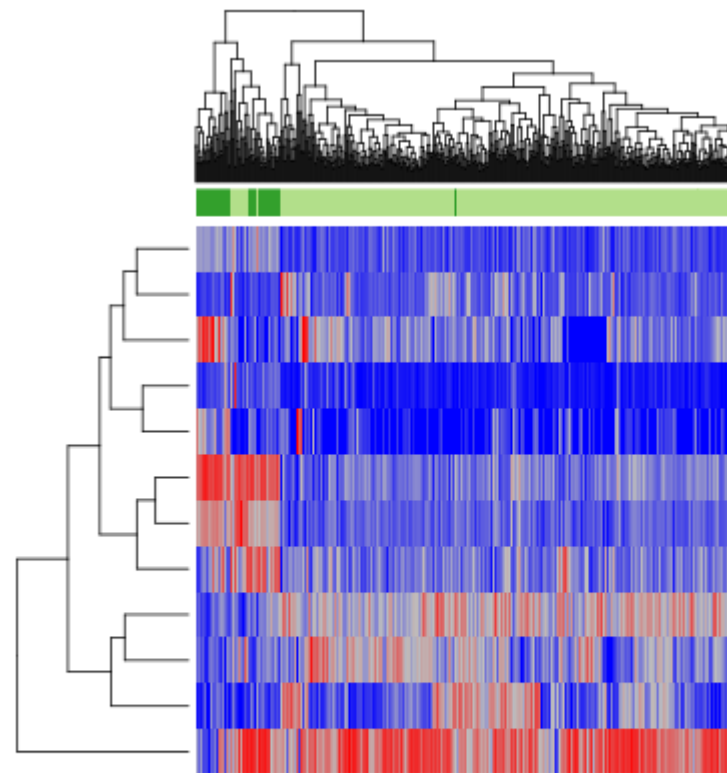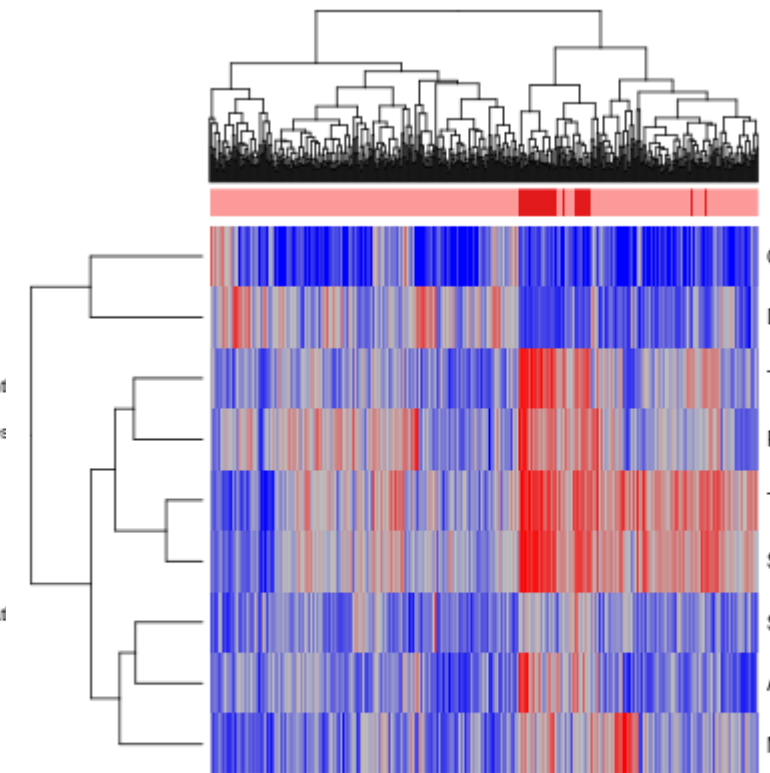

Supplement: Supplementary file 4 — Additional file 4: Figure SF4. Heatmaps of function activation for the three cancers. Description: Heatmaps of function activations for the three cancers. Samples and functions were ordered following the results of a non-supervised hierarchical clusterization. Top row corresponds to Gene Ontology functions and bottom row to Uniprot keywords. Each column represents a cancer, from left to right: breast, kidney and lung cancers. [file 13062_2021_293_MOESM4_ESM.pdf]

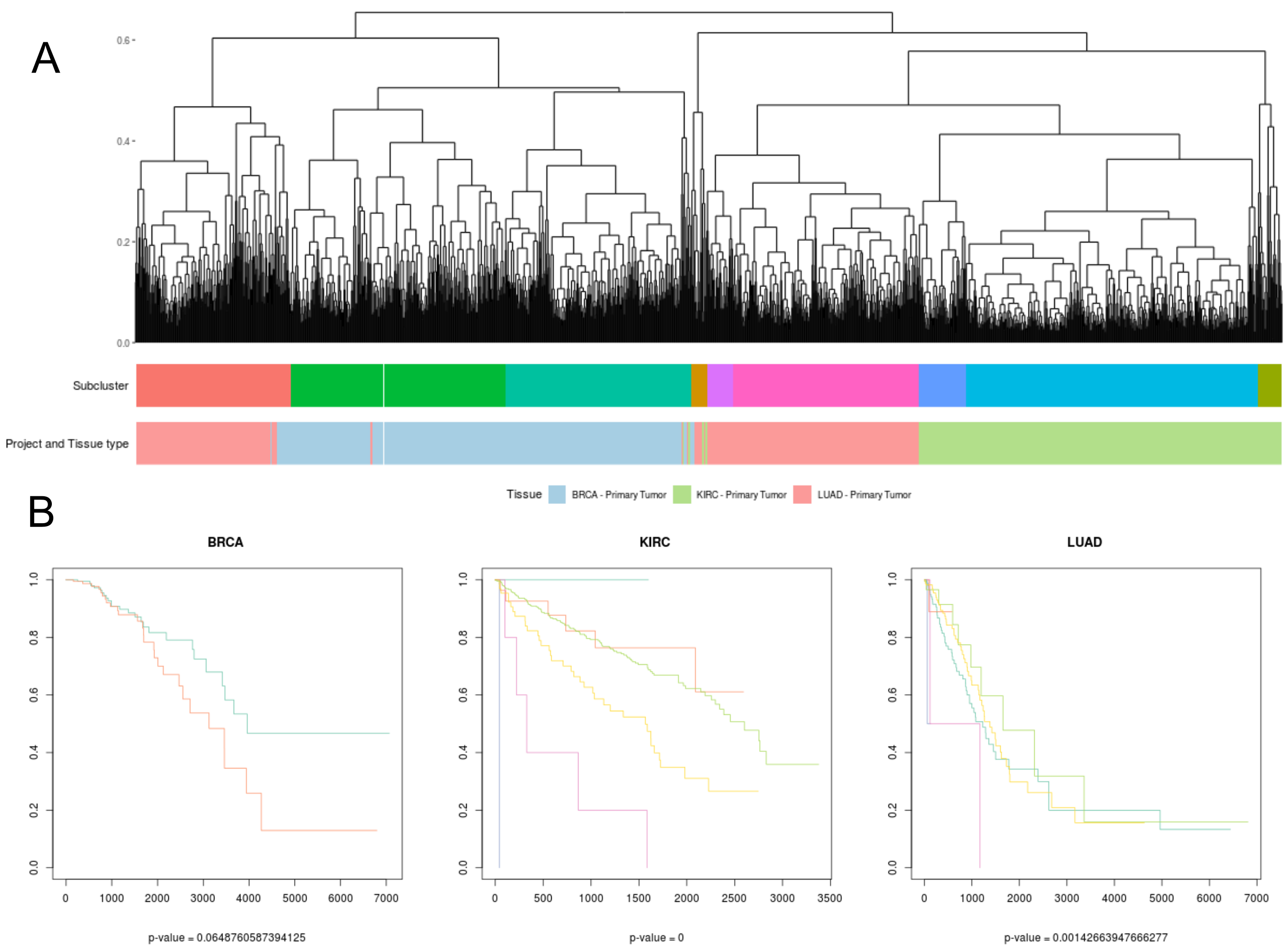

Supplement: Supplementary file 5 — Additional file 5: Figure SF5. Survival analysis of cancer subtypes resulting from clustering by GO terms values. Description: A: Clustering of the tumor samples from BRCA, KIRC and LUAD based on the values of the GO terms which resulted significant in the comparison between healthy and tumor tissues in all three cancer types, colored by their tissue of origin (Tissue) and the subcluster in which they have been stratified. B: Kaplan-Meier curves of the subgroups created in each cancer, with the p-value of the survival analysis performed at the bottom. Curve colors are not matched with the subcluster colors but defined to be easily differentiated. Figure SF6. Survival analysis of cancer subtypes resulting from clustering by Uniprot function values. Description: A: Clustering of the tumor samples from BRCA, KIRC and LUAD based on the values of the Uniprot keywords which resulted significant in the comparison between healthy and tumor tissues in all three cancer types, colored by their tissue of origin (Tissue) and the subcluster in which they have been stratified. B: Kaplan-Meier curves of the subgroups created in each cancer, with the p-value of the survival analysis performed at the bottom. Curve colors are not matched with the subcluster colors but defined to be easily differentiated. Figure SF7. Specific functions per cancer and survival related to AMPK signaling pathway: CCNA2. Description: A) UpSet plot indicating the number of paths in the pairwise intersections among the three analyzed cancers, and the (null) intersection of the three of them. C) Kaplan-Meier curves for the three groups of activation intensity defined by path AMPK signaling pathway: CCNA2 in KIRC (top) and LUAD (bottom). Blue lines correspond to the 20% of samples with lowest activity values, red lines correspond to the 20% of samples with highest activity values of this pathway and orange lines correspond to the remaining 60% of samples. [file 13062_2021_293_MOESM5_ESM.zip › SF5_subclustering_GOsR1.png]

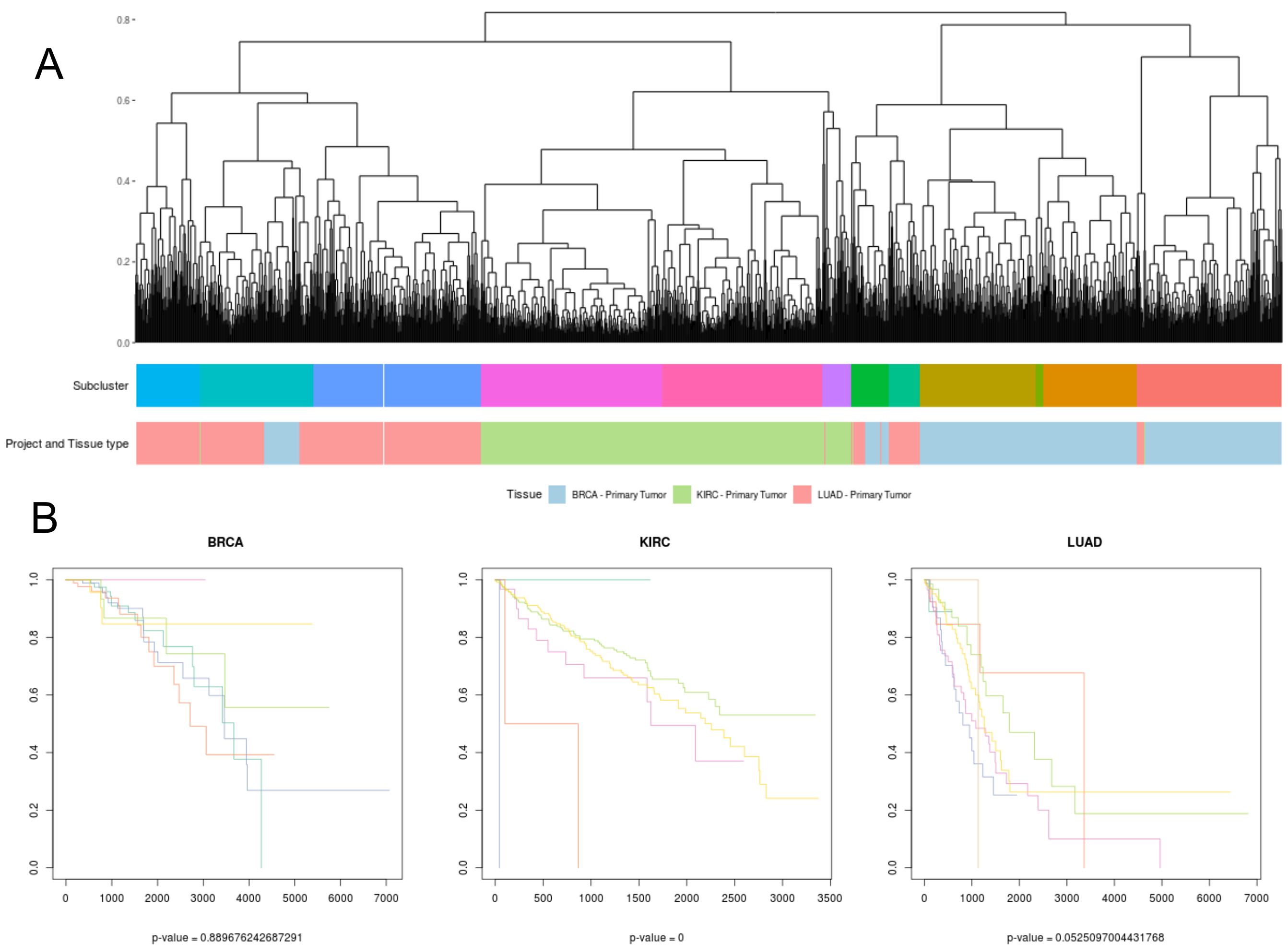

Supplement: Supplementary file 5 — Additional file 5: Figure SF5. Survival analysis of cancer subtypes resulting from clustering by GO terms values. Description: A: Clustering of the tumor samples from BRCA, KIRC and LUAD based on the values of the GO terms which resulted significant in the comparison between healthy and tumor tissues in all three cancer types, colored by their tissue of origin (Tissue) and the subcluster in which they have been stratified. B: Kaplan-Meier curves of the subgroups created in each cancer, with the p-value of the survival analysis performed at the bottom. Curve colors are not matched with the subcluster colors but defined to be easily differentiated. Figure SF6. Survival analysis of cancer subtypes resulting from clustering by Uniprot function values. Description: A: Clustering of the tumor samples from BRCA, KIRC and LUAD based on the values of the Uniprot keywords which resulted significant in the comparison between healthy and tumor tissues in all three cancer types, colored by their tissue of origin (Tissue) and the subcluster in which they have been stratified. B: Kaplan-Meier curves of the subgroups created in each cancer, with the p-value of the survival analysis performed at the bottom. Curve colors are not matched with the subcluster colors but defined to be easily differentiated. Figure SF7. Specific functions per cancer and survival related to AMPK signaling pathway: CCNA2. Description: A) UpSet plot indicating the number of paths in the pairwise intersections among the three analyzed cancers, and the (null) intersection of the three of them. C) Kaplan-Meier curves for the three groups of activation intensity defined by path AMPK signaling pathway: CCNA2 in KIRC (top) and LUAD (bottom). Blue lines correspond to the 20% of samples with lowest activity values, red lines correspond to the 20% of samples with highest activity values of this pathway and orange lines correspond to the remaining 60% of samples. [file 13062_2021_293_MOESM5_ESM.zip › SF6_subclustering_uniprotsR1.png]

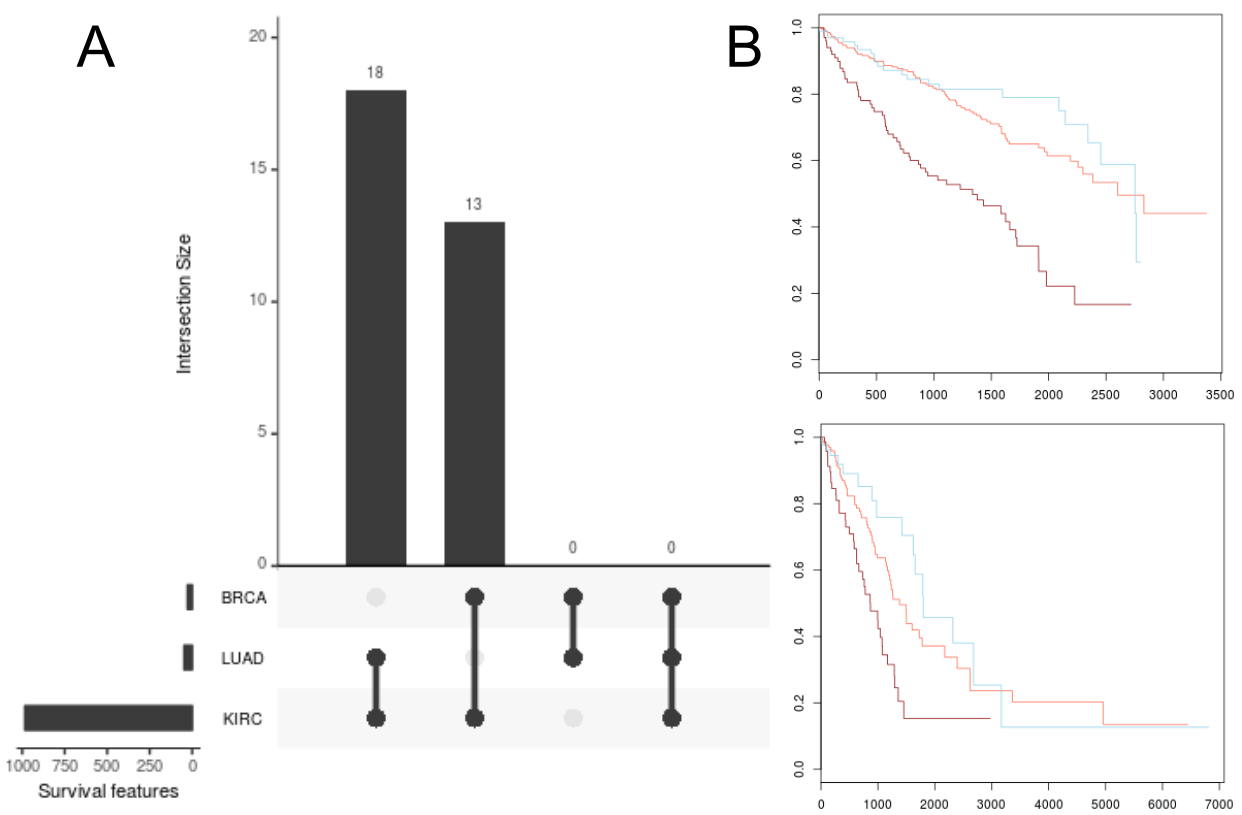

Supplement: Supplementary file 5 — Additional file 5: Figure SF5. Survival analysis of cancer subtypes resulting from clustering by GO terms values. Description: A: Clustering of the tumor samples from BRCA, KIRC and LUAD based on the values of the GO terms which resulted significant in the comparison between healthy and tumor tissues in all three cancer types, colored by their tissue of origin (Tissue) and the subcluster in which they have been stratified. B: Kaplan-Meier curves of the subgroups created in each cancer, with the p-value of the survival analysis performed at the bottom. Curve colors are not matched with the subcluster colors but defined to be easily differentiated. Figure SF6. Survival analysis of cancer subtypes resulting from clustering by Uniprot function values. Description: A: Clustering of the tumor samples from BRCA, KIRC and LUAD based on the values of the Uniprot keywords which resulted significant in the comparison between healthy and tumor tissues in all three cancer types, colored by their tissue of origin (Tissue) and the subcluster in which they have been stratified. B: Kaplan-Meier curves of the subgroups created in each cancer, with the p-value of the survival analysis performed at the bottom. Curve colors are not matched with the subcluster colors but defined to be easily differentiated. Figure SF7. Specific functions per cancer and survival related to AMPK signaling pathway: CCNA2. Description: A) UpSet plot indicating the number of paths in the pairwise intersections among the three analyzed cancers, and the (null) intersection of the three of them. C) Kaplan-Meier curves for the three groups of activation intensity defined by path AMPK signaling pathway: CCNA2 in KIRC (top) and LUAD (bottom). Blue lines correspond to the 20% of samples with lowest activity values, red lines correspond to the 20% of samples with highest activity values of this pathway and orange lines correspond to the remaining 60% of samples. [file 13062_2021_293_MOESM5_ESM.zip › SF7_survivalR1.png]
